# Early Results of PreserFlo MicroShunt Implantation in Complicated Cases: A Single-Center Case Series

**DOI:** 10.7759/cureus.83116

**Published:** 2025-04-28

**Authors:** Nur Fatihin Samiyah Mohamad Hisham, Othmaliza Othman, Norshamsiah Md Din, Rupini Yogesvaran

**Affiliations:** 1 Ophthalmology, Universiti Kebangsaan Malaysia Medical Centre, Kuala Lumpur, MYS; 2 Ophthalmology, Hospital Kuala Lumpur, Kuala Lumpur, MYS

**Keywords:** complication, early result, glaucoma, intraocular pressure, minimally invasive glaucoma surgery, preserflo microshunt

## Abstract

Purpose: We report the initial outcomes, early complications, and management of PreserFlo MicroShunt (Santen Pharmaceutical Co., Ltd., Osaka, Japan) implantation in complicated glaucoma cases.

Methods: This was a retrospective study.

Results: A case series of 12 patients with various glaucoma etiologies underwent PreserFlo MicroShunt surgery at Hospital Canselor Tuanku Muhriz, Kuala Lumpur, Malaysia, from September 2022 to June 2023. Best-corrected visual acuity (BCVA), intraocular pressure (IOP), IOP-lowering medications, and postoperative complications were assessed. All procedures were augmented with intraoperative mitomycin C (0.2 mg/mL). Among the 12 patients (eight males, four females), five (42%) had primary open-angle glaucoma (OAG), five (42%) had secondary OAG, and one (8%) each had ocular hypertension and primary angle closure. Postoperatively, all patients except one showed significant IOP and medication reduction. Mean IOP decreased from 23.75 ± 8.82 to 15.67 ± 10.18 mmHg (34% reduction, p < 0.001), and mean IOP-lowering medications reduced from 4.58 to 0.08 (98% reduction, p < 0.001). Complications included wipeout syndrome due to hypotony (8%) and overfiltration hypotony (16%), with one requiring surgical revision and later a glaucoma drainage device.

Conclusion: Our early results suggest PreserFlo MicroShunt is effective in lowering IOP and medications, but is associated with complications.

## Introduction

Glaucoma is a progressive optic neuropathy that causes the death of the ganglion cell layer of the retina with subsequent changes of the optic nerve head, such as cupping and thinning, both of which cause visual field impairment [1-2]. It is primarily attributed to an abrupt increase and/or prolonged periods of elevated intraoperative pressure (IOP). Glaucoma is the major contributor to blindness worldwide, mainly affecting women and the Asian population [3]. Thus, efforts in developing innovative and effective surgical interventions for glaucoma are essential in providing better treatment options, improving outcomes, and ultimately preserving the vision of individuals affected by this condition.

Surgery is indicated when a satisfactory IOP is not attained by IOP-lowering medication [1]. Micro-invasive glaucoma surgery (MIGS), which is less invasive and less traumatizing, is now gaining popularity and becoming a surgical choice in various types of glaucoma [4]. With MIGS procedures, surgeons are hoping that it could minimize intraoperative and postoperative complications, as well as provide a less traumatizing method of lowering IOP than standard glaucoma surgery, with the objective of minimizing or perhaps stopping topical anti-glaucoma medication [5]. Preserflo MicroShunt (Santen Pharmaceutical Co., Ltd., Osaka, Japan) implantation is the latest microinvasive bleb surgery to be introduced in Malaysia since late 2022 by Santen Pharmaceutical. The PreserFlo MicroShunt is manufactured from isobutylene-block-styrene, which is an extremely inert material that does not produce harm to human tissue. The lumen is made with an optimum size, just enough to prevent overflow of the aqueous humor, and it does not get easily blocked by cells and pigments. The Preserflo length is 8.5 mm, which the segment is further divided by a fin, which is 1 mm, into distal (3 mm) and proximal (4.5 mm) segments. The exterior diameter is 350 μm, and the interior opening is 70 μm. The tip of the device is beveled [6]. It is implanted ab externo, where its distal part is placed underneath a newly formed bleb with the application of mitomycin C under the conjunctiva and Tenon’s capsule. It can be implanted as a standalone procedure or in combination with cataract surgery.

These surgeries have their unique potential in reducing IOP with less trauma and are safer as compared to conventional glaucoma filtering surgery [7-8]. We aim to report the initial outcome, early complications, and their management in a group of complicated glaucoma patients implanted with PreserFlo MicroShunt at our tertiary center.

## Materials and methods

This is a retrospective case series studying the effectiveness, success rate, and complications of the PreserFlo MicroShunt. This case series describes 12 patients with various types of glaucoma who had uncontrolled IOP and showed signs of disease progression despite maximum medical treatment and underwent PreserFlo MicroShunt surgery at Hospital Canselor Tuanku Mukhriz, Kuala Lumpur, Malaysia, from September 2022 to June 2023. Best-corrected visual acuity (BCVA), preoperative and postoperative IOP, number of IOP-lowering medications, and postoperative complications were assessed. Of the 12 patients, five had advanced primary open-angle glaucoma (OAG), five had secondary glaucoma (pseudoexfoliative glaucoma, anterior uveitis with steroid-induced glaucoma, and silicone oil-induced glaucoma), one patient had ocular hypertension, and one patient had primary angle closure. 

Data were collected six times during follow-up visits, on day one, subsequently after seven days, then two weeks, three weeks, one month, and three months. Fortunately, despite the small sample size, no dropouts were recorded. Visual acuity was measured using the Snellen chart and converted into logMAR, anterior segment examination was done using the slit lamp, and IOP was measured using the Goldmann applanation tonometer by the same person that was involved in this study. Postoperatively, visual acuity, IOP, and the number of IOP-lowering medications were recorded. Statistical analysis was performed using IBM SPSS Statistics software version 29.0.1.0 (IBM Corp., Armonk, NY). Continuous data were summarized using mean and standard deviation (SD), while categorical data were presented as frequencies and percentages. The Shapiro-Wilk test was employed to assess the normality of continuous variables.

PreserFlo MicroShunt procedure

Sub-Tenon’s anesthesia is the preferred local anesthesia for all of our patients who undergo Preserflo implantation, as it reduces the anesthesia-related complications as compared to peribulbar and retrobulbar blocks. A fornix-approached conjunctival periotomy was made in the superonasal or superotemporal quadrant, and Tenon's capsule was dissected over an area as wide and posterior as possible. Hemostasis was secured with wet cautery, and mitomycin C-soaked sponges with a concentration of 0.02%, equal to 0.2 mg/ml, were applied underneath the dissected conjunctiva for three minutes. After the sponges were removed, the area was copiously rinsed with 200 mL of balanced salt solution. Using a caliper, we marked the sclera 3 mm from the limbus, and a partial-thickness scleral pocket was made using a 15-degree blade. Then we introduced a 25-gauge needle into this sclera pocket to create an optimum-sized track into the AC. The device was then inserted into the AC through the needle track, with the fin tucked tightly within the pocket. Finally, the aqueous flow from the device is checked, and we ended the procedure by suturing the Tenon’s capsule and conjunctiva and ensuring that it is watertight. The procedures were concluded with a prophylactic intracameral cefuroxime injection and a subconjunctival dexamethasone injection. All cases were done by one surgeon.

Postoperative management

After the surgery, all the IOP-lowering agents were discontinued immediately. Postoperatively, topical ciprofloxacin 0.3% and topical dexamethasone 0.1% were prescribed two hourly for four weeks. Dexamethasone was slowly tapered over several months, depending on the bleb morphology and wound healing. IOP was recorded at every postoperative visit. Topical antiglaucoma medication was added in case of increased IOP.

When necessary, bleb needling was carried out postoperatively under the microscope using a 28-gauge needle attached to a syringe, aimed at disrupting the fibrous capsule surrounding the distal portion of the PreserFlo MicroShunt to reestablish aqueous humor outflow. In cases involving significant fibrosis, a 0.1 ml injection of an antimetabolite agent, 5-fluorouracil (5-FU, 50 mg/ml), was administered beneath the conjunctiva at the end of the procedure.

Statistical analysis

As shown in Table 1, 12 eyes of 12 patients, eight males (66.7%) and four females (33.3%), were included in the study. The mean IOP reduced from 23.75 ± 8.82 mmHg at baseline to 15.67 ± 10.18 mmHg (34 % reduction, p < 0.001) at three months, except for the patient with silicon-induced OAG. The IOP-lowering medications reduced from 4.58 ± 0.793 to 0.08 ± 0.289 (98% reduction, p < 0.001).

**Table 1:** Summary of demographic characteristics and intraocular pressure (IOP) measurements preoperatively and at three months postoperatively

Demographics and baseline characteristics	Overall population (n = 12)
Age, years (mean ± SD)	56.83 ± 19.2
Age range	27–85
Male, n(%)	8 (66.7)
Medicated IOP, mmHg (mean ± SD)	23.75 ± 8.8
Range IOP	14-46
Preoperative IOP ≥ 18 and ≤ 21 mmHg, n (%)	2 (16.7)
Preoperative IOP > 21 mmHg, n (%)	7 (58.3)
Postoperative IOP at 3 months	15.67 ± 10.18
Number of glaucoma medications (mean ± SD)	
Preoperative	4.58 ± 0.79
Range	3-6
Postoperative	0.08 ± 0.289

## Results

**Figure 1:**
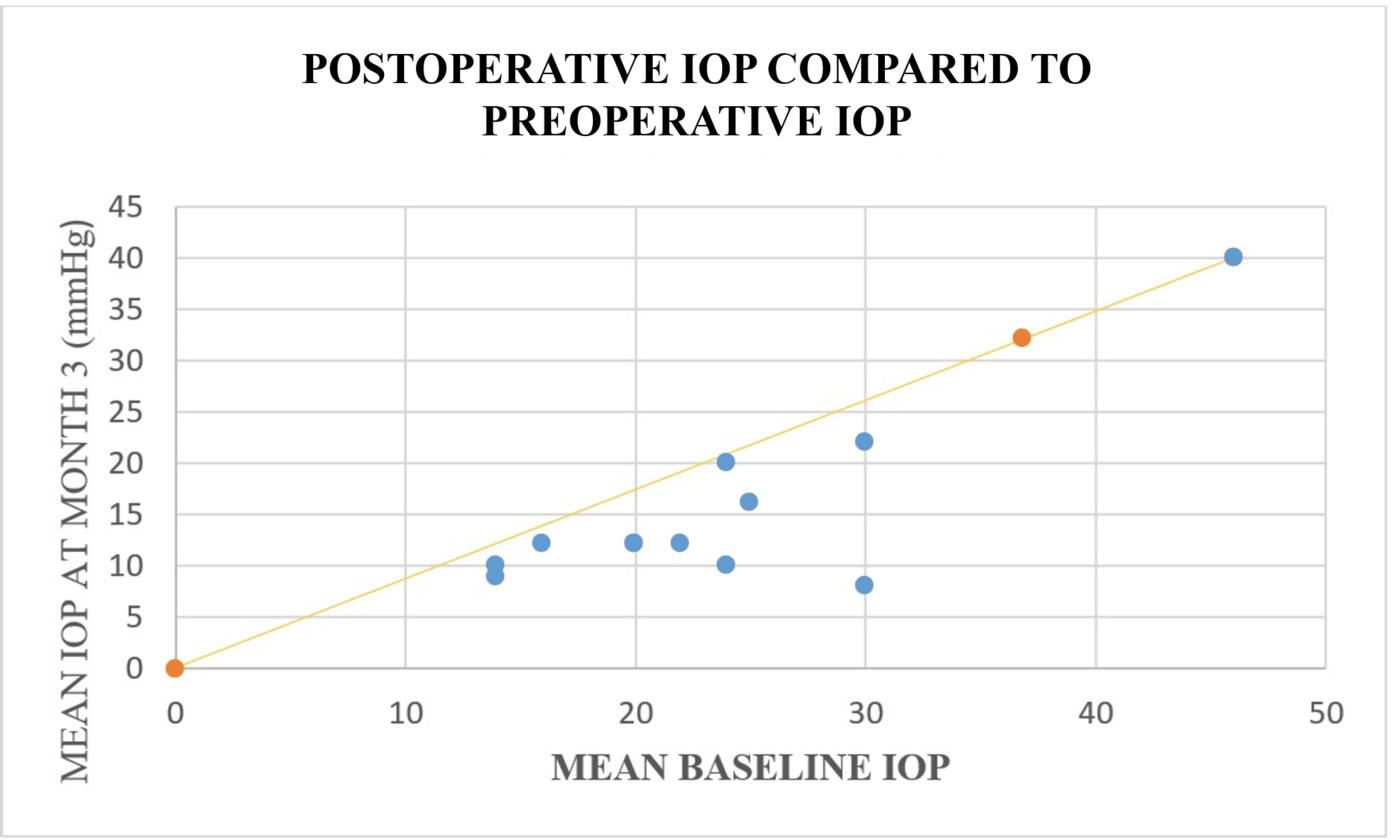
Postoperative intraocular pressure (IOP) levels were compared with preoperative IOP at the three-month follow-up. The diagonal yellow line represents the 20% reduction in mean IOP. The blue dots represent postoperative IOP. This reduction was statistically significant (p < 0.001).

As shown in Figure 1, a significant reduction in IOP was observed following surgery. The mean preoperative IOP was 23.75 mmHg (95% CI: 18.14-29.36), while the mean IOP at three months postoperatively was 15.25 mmHg (95% CI: 9.60-20.90). Both values were significantly greater than 0 (p < 0.001). This represents a mean reduction of 8.5 mmHg, indicating a substantial and clinically meaningful decline in IOP over the three-month postoperative period. Each point represents an individual participant's data, with the trend line indicating the overall relationship. The data reveal a notable reduction in mean IOP three months following surgery. This is visually emphasized by the trend line, which captures the overall downward shift in IOP values. The scatter plot highlights not only the individual variations but also the collective effectiveness of the surgical intervention, as most points fall below the 20% reduction line. These findings suggest a significant improvement in IOP management for the majority of participants.

**Figure 2:**
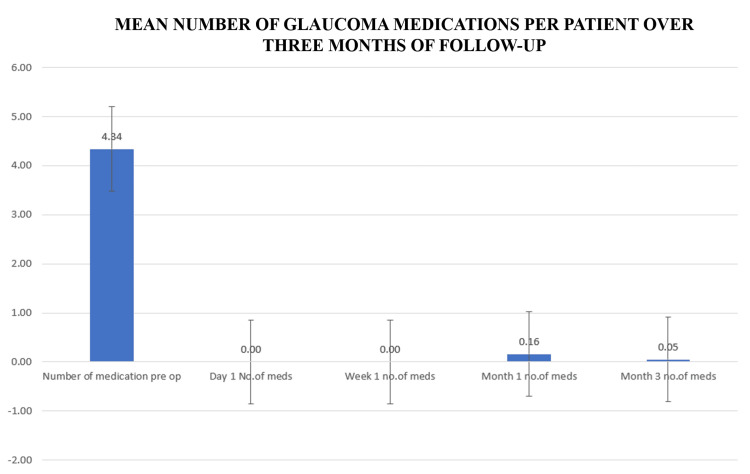
Mean number of glaucoma medications over three months of follow-up The bar chart shows a significant reduction in the number (no.) of medications from baseline (4.34 ± 0.79) to 0.05 ± 0.29 at three months post surgery with a p-value < 0.001, which is statistically significant.

A significant reduction in the number of glaucoma medications was observed over the three-month postoperative follow-up period. At baseline, patients were using an average of 4.34 ± 0.79 topical glaucoma medications. By three months after surgery, this number had markedly decreased to 0.05 ± 0.29, reflecting a near-complete cessation of pharmacologic therapy. This reduction was statistically significant (p < 0.001), indicating a substantial decrease in medication burden following the surgical intervention. The findings are illustrated in Figure 2, which highlights the progressive decline in medication use postoperatively.

Table 2 below compares the preoperative and postoperative logMAR of each patient.

**Table 2:** Visual acuity of 12 patients before and after the surgery measured using the logMAR scale. Negative values in the "Change" column indicate improvement in vision, while positive values indicate deterioration. One patient demonstrated a significant loss of vision postoperatively (from logMAR 0.3 to 4.0), potentially indicative of wipe-out syndrome.

Preoperative logMAR	Postoperative logMAR	Change (post-pre)
0.5	0.3	-0.2
0.8	0.8	0
0.3	4	3.7
1.8	1.8	0
0.3	0.3	0
0.2	0.2	0
0.6	0.6	-0.33
0.63	0.3	-0.8
1	0.2	0
1.5	1.5	0
0.5	0.477	-0.023
1	0.477	-0.523

A paired samples t-test was conducted to assess the potential occurrence of wipe-out syndrome [9] following ocular surgery by comparing preoperative and postoperative logMAR scores of the 12 patients. The mean difference in logMAR scores was -0.152 (SD = 1.147), where negative values indicate a trend toward improved visual acuity. However, this difference was not statistically significant, t(11) = -0.459, p = 0.655. The 95% confidence interval for the mean difference ranged from -0.881 to 0.577, indicating considerable variability and including the null value.

These findings suggest that, at the group level, there was no significant deterioration in visual acuity following surgery, and therefore, no statistical evidence to support the occurrence of wipe-out syndrome within this study.

Early postoperative complications

One patient (8%) had wipe-out syndrome due to clinical hypotony from ciliary shutdown, two patients (16%) had numerical hypotony due to overfiltration that resolved spontaneously, and one patient (8%) required surgical revision and additional glaucoma drainage device implantation due to suboptimal IOP control.

The patient with hypotony is a 66-year-old lady with right-eye steroid-induced glaucoma and uncontrolled IOP despite maximum tolerated medical therapy (MTMT). She had a history of acute anterior uveitis due to sarcoidosis. She had undergone trabeculectomy in 2020 but failed. Her vision prior to Preserflo MicroShunt implantation was right eye (OD) 3/60 and left eye (OS) 6/9. The cup-to-disc (CDR) ratio of the right eye was 0.95. Preserflo MicroShunt was commenced on 21^st^ November 2022. Postoperatively, she developed hypotony with IOP 2-5 mmHg with maculopathy. Subsequently, her IOP picked up within three weeks with the resolution of maculopathy. Her current IOP ranges between 8 mmHg and 10 mmHg with improved vision to BCVA of 6/24 in the right eye.

The patient with hypotony due to overfiltration was a 28-year-old Chinese lady with underlying ocular hypertension in her right eye and had undergone multiple glaucoma surgeries, which included Xec tube (AbbVie Inc., North Chicago, IL) implantation in June 2020 and augmented trabeculectomy in March 2021 before the Preserflo MicroShunt procedure. Despite multiple needling procedures, her IOP remained suboptimal. She had the Preserflo MicroShunt implanted on 14^th^ November 2022, 20 months after the failed trabeculectomy. The PreserFlo MicroShunt implantation procedure was uneventful. On postoperative day one, a leaked conjunctival wound was noted, and her IOP was 5 mmHg. A bandage contact lens was applied, and no further leak was seen after three days. However, the bleb was diffuse and dome-shaped, with unrecordable IOP, indicating overfiltration. A torpedo pad was applied, and her IOP picked up to 10 mmHg the next day. The bleb remained diffuse and raised. Subsequent reviews indicated a well-controlled IOP ranging between 10 and 12 mmHg.

The case with hypotony with ciliary shutdown and wipe-out syndrome was a 72-year-old Malay male patient with underlying hypertension, ischemic heart disease, rheumatoid arthritis, and a history of lacunar infarct who underwent right eye Preserflo MicroShunt procedure for pseudoexfoliative glaucoma. His right eye previously underwent selective laser trabeculoplasty and cataract surgery. He was on maximum tolerated medical therapy prior to surgery with a BCVA of 6/24. The procedure was uneventful. On day one postoperatively, his IOP was 6 mmHg, the anterior chamber (AC) was deep, and the bleb was flat. His vision after one week dropped to only light perception, but the AC was still deep. Viscoelastic was injected into the AC to bring up the pressure to 15 mmHg. The next day, his IOP shot up to 54 mmHg with corneal bedewing and vision of hand movement. AC paracentesis was done to remove the viscoelastic device. Unfortunately, he developed wiped-out syndrome with no perception of light (NPL) after one month postoperatively, with a vision of NPL and IOP of 07 mmHg. He was treated conservatively.

The patient who required a secondary glaucoma procedure was a 37-year-old lady with high myopia of -9.00D. She had a history of right eye vitrectomy and phacoemulsification without lens implantation for rhegmatogenous retinal detachment involving the macula. Two weeks post surgery, she had persistently high IOP requiring maximum IOP-lowering medications. Upon presentation, there was no relative afferent pupillary defect; her vision was counting fingers OD and 6/6 OS, with IOP of 46 mmHg OD and 19 mmHg OS. Her right conjunctiva was injected, corneal bedewing was seen, the AC was deep, the angles were open, and no silicone oil was seen in the AC or angles. The retina was flat with an oil-filled vitreous cavity. The CDR ratio was 0.5 OU. Preserflo MicroShunt implantation was the chosen procedure due to the recent vitrectomy, and the port sites were still not fully healed. Intraoperatively, after initial inadvertent entry into the sulcus, the needle tract was made into the AC, and the rest of the surgery was unremarkable. Unfortunately, at one week postoperatively, a vitreous strand was seen at the lumen of the tube, causing elevated IOP to 40 mmHg. After yttrium-aluminum-garnet (YAG) vitreolysis, flow was restored, and IOP reduced to 10 mmHg. IOP began to shoot up again in the second week postoperatively, and needling of the tube was performed with subconjunctival 5-FU and dexamethasone injection. Needling was repeated again at four and five weeks postoperatively due to bleb fibrosis, and IOP-lowering medications were restarted as IOP was 47 mmHg. Revision of PreserFlo MicroShunt was done at two months postoperatively, but the silicone oil started to migrate to the AC on day 1 post revision, although it was not blocking the tube. Unfortunately, silicone oil then migrated to the pupillary margin, and IOP shot up to 51 mmHg. Although the silicone oil was finally removed, the IOP showed an increasing trend, ranging between 25 and 45 mmHg while on maximum IOP-lowering medications. Despite multiple needling procedures and bleb revision, we were unable to sustain a favorable IOP, and a glaucoma drainage device was implanted.

## Discussion

The introduction of MIGS has brought significant advancements in the treatment of glaucoma, aiming to achieve effective IOP reduction with minimal invasiveness and a reduced reliance on topical medications. In this study, we reported the preliminary outcomes, success rates, and complications of PreserFlo MicroShunt implantation in a cohort of 12 patients with various etiologies of glaucoma. We did not compare the results of different mitomycin C concentrations, as we used 0.2 mg/ml in all of our patients, although some studies show that the concentration and duration of mitomycin C exposure affect the surgical success of glaucoma filtration surgery [10]. All patients’ IOP levels were significantly reduced after the first follow-up and remained stable throughout the subsequent follow-ups.

A landmark study comparing MicroShunt with trabeculectomy had recently been published [11,12]. The most common complications were either an increase in IOP or hypotony, bleb leak, and subconjunctival bleed, which would be expected to be lower compared to the trabeculectomy group. The more serious sight-threatening complication, which includes malignant glaucoma, choroidal effusion requiring surgical intervention, and IOP spike > 10 mmHg from baseline, was low in both groups, which was 1.0% in the MicroShunt and 0.8% in the trabeculectomy group. Now, the Preserflo MicroShunt is gaining popularity as a minimally invasive glaucoma procedure with its good efficacy in reducing IOP and safety profile compared to other MIGS [6,13,14].

IOP reduction and medication reduction

The results of our study demonstrate a notable reduction in mean IOP from baseline to three months post surgery. This reduction was statistically significant (p < 0.001) and clinically relevant, with a 34% decrease in mean IOP. These findings are consistent with the primary objective of glaucoma management, which is to lower IOP to prevent optic nerve damage. Importantly, the reduction in IOP was achieved across different types of glaucoma, including primary OAG, secondary OAG, ocular hypertension, and primary angle-closure.

Furthermore, the reduction in the number of glaucoma medications was substantial, with a 98% decrease from baseline. This is a significant benefit for patients, as it not only reduces the financial burden of medications but also improves adherence and reduces the potential side effects associated with long-term medication use.

Complications and management

While the PreserFlo MicroShunt procedure showed promising IOP control and medication reduction, it is essential to acknowledge the observed complications. One patient (8%) experienced wipe-out syndrome due to hypotony resulting from ciliary shutdown. This underscores the importance of careful patient selection, postoperative IOP monitoring, and taking measures to prevent excessively low IOP, which can be detrimental to vision.

Additionally, two patients (16%) experienced hypotony due to overfiltration. Fortunately, these cases resolved spontaneously, highlighting the dynamic nature of postoperative IOP control in MIGS procedures. It is crucial for clinicians to be vigilant in managing early postoperative hypotony to avoid potential complications.

In one case (8%), surgical revision and additional glaucoma drainage device implantation were required due to suboptimal IOP control. This highlights the need for ongoing assessment and potential interventions in cases where the desired IOP reduction is not achieved.

Limitations and future directions

This study has its own limitations, including its retrospective nature, the relatively small sample size, and the limited follow-up period of three months. Longer follow-up and a larger cohort would provide a more comprehensive understanding of the sustained efficacy and safety profile of PreserFlo MicroShunt implantation. Additionally, the inclusion of a control group would facilitate a direct comparison with standard glaucoma management approaches. Long-term evaluations are essential in ascertaining long-term surgical safety and efficacy, as recent studies have shown some increased failure rates beyond six months [7].

## Conclusions

In conclusion, the early results of PreserFlo MicroShunt implantation in our study demonstrate promising outcomes in terms of IOP control and satisfactory reduction in antiglaucoma medication. However, the occurrence of complications underscores the importance of vigilant postoperative monitoring and timely intervention when necessary. Further research with larger cohorts and longer follow-up periods is warranted to assess the durability of these outcomes and to refine the selection criteria for this micro-invasive glaucoma surgery technique. PreserFlo MicroShunt implantation holds potential as a valuable addition to the armamentarium of glaucoma treatment options, offering a minimally invasive approach to IOP control.
